# Elevated Levels of Serum Biomarkers Associated with Damage to the CNS Neurons and Endothelial Cells Are Linked with Changes in Brain Connectivity in Breast Cancer Patients with Vestibulo-Atactic Syndrome

**DOI:** 10.3390/pathophysiology30020022

**Published:** 2023-06-15

**Authors:** Alexandra Nikolaeva, Maria Pospelova, Varvara Krasnikova, Albina Makhanova, Samvel Tonyan, Yurii Krasnopeev, Evgeniya Kayumova, Elena Vasilieva, Aleksandr Efimtsev, Anatoliy Levchuk, Gennadiy Trufanov, Mark Voynov, Maxim Shevtsov

**Affiliations:** 1 Personalized Medicine Centre, Almazov National Medical Research Centre, Akkuratova Str. 2, 197341 Saint Petersburg, Russia; pospelovaml@mail.ru (M.P.); krasnikova_vv@almazovcentre.ru (V.K.); a.mahanova.a@mail.ru (A.M.); samvelium@gmail.com (S.T.); krasnopeev.medicina@mail.ru (Y.K.); kayumova_ee@almzovcentre.ru (E.K.); atralf@mail.ru (A.E.); feuerlag999@yandex.ru (A.L.); trufanovge@mail.ru (G.T.); m.vojnov@mail.ru (M.V.); 2 Department of Radiation Oncology, Technishe Universität München (TUM), Klinikum rechts der Isar, Ismaninger Str. 22, 81675 Munich, Germany

**Keywords:** breast cancer, vestibulo-atactic syndrome, functional MRI, ICAM-1, PECAM-1, NSE, NR-2-ab, neuroimaging, connectome, cognitive impairment, quality of life

## Abstract

Vestibulo-atactic syndrome (VAS), which represents a combination of motor and vestibular disorders, can be manifested as a clinical complication of breast cancer treatment and has a significant impact on patients’ quality of life. The identification of novel potential biomarkers that might help to predict the onset of VAS and its progression could improve the management of this group of patients. In the current study, the levels of intercellular cell adhesion molecule 1 (ICAM-1), platelet/endothelial cell adhesion molecule 1 (PECAM-1), NSE (neuron-specific enolase), and the antibodies recognizing NR-2 subunit of NMDA receptor (NR-2-ab) were measured in the blood serum of BC survivor patients with vestibulo-atactic syndrome (VAS) and associated with the brain connectome data obtained via functional magnetic resonance imaging (fMRI) studies. A total of 21 patients were registered in this open, single-center trial and compared to age-matched healthy female volunteers (control group) (*n* = 17). BC patients with VAS demonstrated higher serum levels of ICAM-1, PECAM-1, and NSE and a lower value of NR-2-ab, with values of 654.7 ± 184.8, 115.3 ± 37.03, 49.9 ± 103.9, and 0.5 ± 0.3 pg/mL, respectively, as compared to the healthy volunteers, with 230.2 ± 44.8, 62.8 ± 15.6, 15.5 ± 6.4, and 1.4 ± 0.7 pg/mL. According to the fMRI data (employing seed-to-voxel and ROI-to-ROI methods), in BC patients with VAS, significant changes were detected in the functional connectivity in the areas involved in the regulation of postural-tonic reflexes, the coordination of movements, and the regulation of balance. In conclusion, the detected elevated levels of serum biomarkers may reveal damage to the CNS neurons and endothelial cells that is, in turn, associated with the change in the brain connectivity in this group of patients.

## 1. Introduction

According to the Global Cancer Observatory (GLOBOCAN), the number of new cases of breast cancer (BC) in 2020 was more than 2.2 million, causing, according to the WHO, 685,000 deaths globally [1,2]. Due to the wide population coverage and the implementation of screening programs that provide an early diagnosis of the disease, the survival prognosis following breast cancer treatment has improved significantly in recent years, with a decrease in mortality of 29%. Currently, the 5-year survival rates are around 90%, and the 10-year survival rates are around 80% [3]. Thus, the life expectancy of patients is increasing, consequently increasing the frequency of long-term side effects associated with the therapy of the underlying disease. Indeed, the number of survivors after radical breast cancer treatment is also growing, as there has been a steep increase in the number of various complications following the therapy. These complications, termed post-mastectomy syndrome, can develop months and years after treatment and affect between 20% and 50% of women [4,5].

Post-mastectomy syndrome is a complex combination of clinical symptoms of organic and functional nature that occurs in patients after a radical mastectomy [6,7,8]. Its occurrence is associated with surgical intervention, leading to reactive inflammation with the development of fibrous and cicatricial changes, followed by compression of the neurovascular bundle, the formation of contractures, and impaired lymphatic drainage [6,9]. The process is further aggravated by subsequent radio- and chemotherapy via the mechanisms of oxidative damage, genetic mutations, alterations in the blood–brain barrier, reduced neuro- and gliogenesis, and neuroinflammation [8,10,11,12,13,14,15].

The main clinical manifestations for breast cancer survivors (BCSs) include postoperative defect on the side of the operation, cicatricial changes [16], plexopathy of the brachial plexus [17,18], impaired biomechanics in the shoulder joint [19], anxiety and depressive disorders [20,21], lymphedema of the upper limb [9,22,23,24], and vestibulo-atactic syndrome (VAS) [25]. Disorders of the central nervous system (CNS) that occur against the background of the complex treatment of breast cancer can lead to functional and structural changes in the brain of patients in the postoperative period, such as changes in functional connectivity in the Default Mode Network and quantitative fractional anisotropy in the white matter tracts [26]. Resting-state fMRI has previously shown alterations in the connectivity of the medial prefrontal cortex in the left hemisphere of the cerebellum in BCS with symptoms of cerebellar ataxia [26,27]. Breast cancer patients treated with cytotoxic agents regularly report cognitive problems. Thus, cognitive deficits have been observed in neuropsychological studies even years after the cessation of chemotherapy. Women complain of concentration problems, difficulties with finding words, and trouble remembering new information [28,29,30].

It has been established that about 15% of oncological patients have cerebrovascular disorders, significantly worsening their condition and prognosis and increasing the level of in-hospital post-stroke mortality. Moreover, patients with ischemic stroke with an active oncological process have a younger age and more pronounced neurological deficit. At the same time, in the postoperative period, mortality rates from cardiovascular diseases occupy a leading position in the structure of mortality among breast cancer patients older than 50 years and account for 35% of total mortalities not directly related to the oncological process [7,31].

According to the literature, BCSs have an increased risk of cardiovascular complications such as acute and chronic, ischemic, and hemorrhagic disorders of cerebral circulation in the carotid and vertebrobasilar basins. These clinical symptoms appear after complex treatment and are not associated with a relapse of the underlying disease or metastatic lesions of the CNS. The development of these manifestations is associated both with the direct toxic effect of chemotherapy and radiation therapy, as well as with the steno-occlusive process in the subclavian artery and its branches with spasmodic scalene muscles and fibro-cicatricial postoperative and post-radiation changes on the side of the surgical intervention [32,33].

Currently, in neuroscience, one of the leading areas of interest is the concept of the brain connectome—a set of functional neural networks and pathways of white matter, the structural and functional changes of which are visualized in various pathological conditions and processes. Functional magnetic resonance imaging (fMRI) allows the possibility of evaluating functional connections in the so-called resting networks of the brain, the most important of which is the network of the passive mode of brain operation (i.e., the medial prefrontal cortex, cortex of the posterior part of the cingulate gyrus, and preclinium), which regulates the flow of cognitive processes including memory, attention, and emotions [34]. Indeed, malfunctioning of the Default Mode Network (DMN), the most significant resting network of the brain, has been demonstrated in many neurological and psycho-organic diseases [35,36,37].

In the current study, we aimed to correlate the clinical manifestation of VAS in BC patients, fMRI data, and the serum levels of biomarkers of CNS and endothelial damage, including ICAM-1 (intercellular adhesion molecule 1), PECAM-1 (platelet/endothelial cell adhesion molecule 1), NSE (neuron-specific enolase), and the level of antibodies recognizing NR-2 subunit of NMDA receptor (NR-2-ab). ICAM-1 is a surface cellular glycoprotein potentially involved in the processes of inflammation, also serving as a biomarker of endothelial dysfunction [38,39]. Thus, several studies have reported a significant increase in the levels of soluble ICAM-1 molecules in patients with vascular cognitive impairment and Alzheimer’s disease, attributing the marker elevation to neuroinflammation and cerebrovascular dysfunction [40,41]. Another molecule of the immunoglobulin superfamily, PECAM-1, also participates in neuroinflammation, showing its prognostic potential in such clinical conditions as stroke and the relapse of multiple sclerosis [42,43,44]. NSE, an isoenzyme of the glycolytic enzyme enolase, can also be employed as a marker for ischemic stroke, intracranial hemorrhage, and traumatic brain injury, indicating changes in neuronal activity [45,46]. In turn, another serum biomarker—antibodies against the NR2 subunit of the NMDA receptor (NR-2-ab)—has been demonstrated to be a potential biomarker for ischemic stroke [47] and cerebral small vessel disease [48]. All of the abovementioned results determined the choice of these molecules for the current study. These molecules are released into the peripheral blood during cerebral injury, both acute and chronic, and can serve as markers of the dynamic process that occurs in the brain, which, in turn, can help to determine the severity of ischemic damage, predict outcomes, and monitor therapeutic effect [49].

Herein, in BC patients with vestibulo-atactic syndrome (*n* = 21), we revealed the correlation between the higher serum levels of ICAM-1, PECAM-1, NSE and the significant changes in the functional connectivity of the brain (according to fMRI data), thus indicating the plausible application of these biomarkers for the evaluation of neuronal and endothelial cell damage in this group of patients.

## 2. Materials and Methods

### 2.1. Patients

An open, single-center, uncontrolled study of functional connectivity and the determination of biomarkers of vascular endothelial and central nervous system lesions in female patients with newly diagnosed manifestations of vestibulo-atactic syndrome and without VAS in the late period of radical treatment of breast cancer was carried out. The study was conducted in accordance with the principles of the Helsinki Declaration of the World Medical Association, with the consent of the Ethics Committee of the Almazov National Medical Research Center (Protocol number 05112019 from 11 November 2019). All patients included in the study signed an informed voluntary consent form.

#### 2.1.1. Inclusion Criteria

The study included 21 breast cancer survivors (14 patients with manifestations of VAS (study group) and 7 patients without VAS (comparison group)) and 17 healthy female volunteers (control group). The inclusion and exclusion criteria of BCS were approved by the Ethics Committee of the Almazov National Medical Research Center (Protocol number 05112019 from 11 November 2019) [25].

The average age of the patients in study group constituted 45.5 ± 5.19 years (age: 31–50 years) and was matched to the VAS− comparison group 45.5 ± 4.1 (age: 30–50 years). The control group included women with no history of oncological and somatic diseases in the stage of decompensation whose average age was 44.25 ± 3.54 years (age: 37–48 years old) (Table 1).

#### 2.1.2. Exclusion Criteria

The exclusion criteria included acute cerebrovascular accident, myocardial infarction, history of traumatic brain injury, advanced oncological diseases, decompensation of chronic diseases, and mental disorders. All patients underwent an MRI of the brain at the beginning of the study to exclude a metastatic process and continued tumor growth.

#### 2.1.3. Neurological Examination

The patients underwent a detailed neurological examination, which included taking an anamnesis (date and extent of surgery, a course of chemotherapy and/or radiation therapy), as well as an assessment of complaints of headaches, dizziness, sleep disturbances, syncope, and drop attacks. Cognitive functions and mental status were assessed (orientation to time, place, and person, short-term and long-term memory, verbal and mathematical abilities, writing, attention, concentration, praxis, and gnosis). Examinations of the cranial nerves I-XII, motor system (movements, tone, strength, muscle bulk, reflexes), sensory system (vibration, joint position sense, light touch, pain, hot/cold), and autonomic nervous system were performed. Vestibular disorders have been studied in more detail. Gait, balance, and coordination were assessed. The Unterberger test was carried out, during which the patients were asked to walk in place with their eyes closed. Then, the Romberg test was carried out, in which the patients were asked to stand erect with their feet together and stretch their arms forward, with their eyes open and then closed. Coordination tests were also carried out (Finger-to-Nose Test, Heel-to-Shin Test, diadochokinesis and dysmetria tests). All of the above tests and methods allowed us to identify the clinical manifestations of VAS in patients.

### 2.2. Evaluation of Plasma Levels of ICAM-1, PECAM-1, NSE, NR-2-ab

The serum (7 mL blood) was sampled from BCS and healthy volunteers’ blood and stored at −70 °C. Quantitative determination of soluble adhesion molecules type 1 intercellular adhesion molecule (ICAM-1), type 1 endothelial and platelet adhesion molecule (PECAM-1), neuron-specific enolase (NSE), and antibodies to destroyed NMDA receptors (NR-2-ab) in blood serum was performed by immunoenzyme analysis using the commercially available ProcartaPlex Human PECAM-1 SIMPLEX kit (both Affymetrix (eBioscience), Santa Clara, CA, USA), ProcartaPlex Human ICAM-1 SIMPLEX kit (both Affymetrix (eBioscience), Santa Clara, CA, USA), kit for the quantitative determination of antibodies to the NR-2 subunit of the NMDA receptor (DRD Biotech, Moscow, Russia), and CanAg NSE EIA kit (Fujirebio, Tokyo, Japan) according to the manufacturers’ protocols.

### 2.3. MR Imaging Protocol

MRI scanning was used as an objective diagnostic method. The study was carried out by MR tomograph with a magnetic field strength of 3.0 T (Siemens, Munich, Germany). Patients underwent structural MRI of the brain using standard pulse sequences (T1-, T2-, TIRM, MPRAGE, DWI), as well as the fMRI technique blood oxygenation level-dependent mode (BOLD). Standard pulse sequences were used to exclude brain pathology DCS and in the control group. The pulse sequence parameters are shown in Table 2.

Functional MRI was used to evaluate the operation of neural networks and their interconnection, employing the BOLD mode. During the study and post-processing, hemodynamically significant changes were visualized that occurred in response to the activation of various areas of the brain, which made possible to assess the functional connectivity of the brain.

The most used variant of functional MRI at the moment is fMRI at rest (rfMRI, resting-state fMRI). The, fMRI data were obtained while participants rested with eyes closed using a T2-weighted gradient echo spiral pulse sequence: TR = 4000 ms, TE = 113 ms, FoV = 22 cm. A high-order shimming method was employed to reduce field heterogeneity. A high-resolution, prepared MPRAGE scan was also acquired and used for spatial normalization of the fMRI: TR: 2300 ms, TE: 2.98 ms, FoV: 25.6 cm, phase FoV: 93.8%, slice thickness: 1.2 mm, 160 slices, and scan time: 5:03.

Functional connectivity preprocessing was performed with Statistical Parametric Mapping v12 and CONN v21a Toolboxes (available online: https://web.conn-toolbox.org/conn-in-pictures (accessed on 19 March 2023)) implemented in MATLAB v2021b (MathWorks, Inc., Natick, MA, USA). Briefly, this involved realignment, co-registration with the segmented anatomic volume, spatial normalization, artifact detection, and smoothing (FWHM = 8 mm), followed by band-pass filtering (0.008–0.09 Hz) and noise correction [50].

### 2.4. Statistics

Statistica 12.5 software (TIBCO Software Inc., Palo Alto, CA, USA) was used for statistical analysis. We used the absolute and relative indicators of the number of observations to assess the qualitative variables, and *p*-values under 0.05 were considered statistically significant. Quantitative variables were represented by medians and ranges of values (Me [25 Percentile; 75 Percentile]).

The normality of the distribution was assessed using the Shapiro–Wilk test. Statistical hypotheses were tested using the Kruskal–Wallis test. Post hoc analysis was performed in pairs using the Mann–Whitney U-test for groups demonstrating a statistically significant result.

## 3. Results

### 3.1. Patients

Patients in the studied group (*n* = 21) had clinical manifestations of VAS: headaches (38%), dizziness (41%), unsteadiness of walking (17%), and syncope (4%).

### 3.2. Assessment of ICAM-1, PECAM-1, NSE, and NR-2-ab

The ICAM-1 serum level in the control group was 230.2 ± 44.8 pg/mL, while in the group with VAS, we detected a nearly three-fold increase in the ICAM-1 level to 654.7 ± 184.8 pg/mL (Table 3). In the group without VAS, the ICAM-1 levels were also increased, with values of 551.9 ± 80.9 pg/mL as compared to the control group.

The PECAM-1 serum level in the control group was 62.8 ± 15.6 pg/mL, while in the group with VAS, we observed a two-fold increase to 115.3 ± 37.03 pg/mL, as well as a slight increase in the group without VAS, with values of 106 ± 36.4 pg/mL.

The NSE serum level in the control group was 15.5 ± 6.4 pg/mL, while in the group with VAS, it was elevated 3.2-fold to 49.9 ± 103.9 pg/mL. In the group without VAS, it was 20.3 ± 15.7 pg/mL.

The NR-2-ab serum level in the control group was 1.4 ± 0.7 pg/mL, while in the group with VAS, it was 0.5 ± 0.3 pg/mL. In the group without VAS, the NR-2-ab serum level was 0.58 ± 0.34 pg/mL (Table 3).

**Figure 1:**
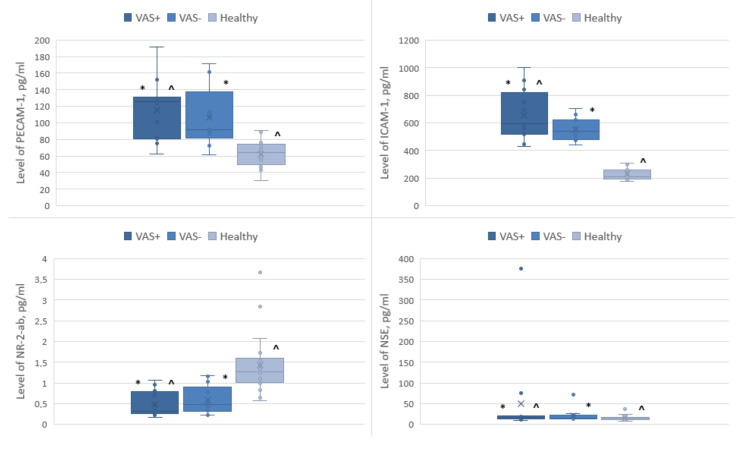
Levels of biomarkers of CNS and endothelial lesions in blood serum *—in comparison with VAS+/VAS−, ^—in comparison with VAS+/control group, ×—median value of a box plot.

Compared with the group of healthy volunteers, patients with VAS had a higher content of ICAM-1, PECAM-1, NSE and a lower value of NR-2-ab (Figure 1).

The differences between the VAS+ and healthy groups were statistically significant for ICAM-1 molecules (*p* < 0.05). In the intergroup comparison of biomarker levels, PECAM-1, NSE, and NR-2 did not show significant differences in any of the studied subgroups.

### 3.3. fMRI Studies

According to the results of fMRI, in patients with VAS, compared to the control group, we observed significant changes in the functional connectivity of the brain (both the strengthening and weakening of connectivity) while performing intergroup statistical analysis (*p* < 0.005) using seed-to-voxel and ROI-to-ROI methods.

In patients with newly diagnosed VAS in the late postoperative period, there was a significant change in the functional connectivity of the brain in the areas involved in the regulation of postural-tonic reflexes, coordination of movements, and regulation of balance.

Patients without VAS, in contrast to the group of patients with VAS, had stronger functional connections between the left occipital lobe, the accessory motor area on the right, and the anterior sections of the fusiform gyrus on the left; between the calcarine fissure on the left, the superior cerebellar peduncle and the posterior lobar cerebellum on the right; between the cerebellar vermis and the flocculus on the left; between the anterior sections of the middle temporal gyrus on the right and between the superior cerebellar peduncle and the posterior lobar cerebellum ipsilaterally; and between the cingulate gyrus, the salience network, and the anterior prefrontal cortex (Table 4).

**Figure 2:**
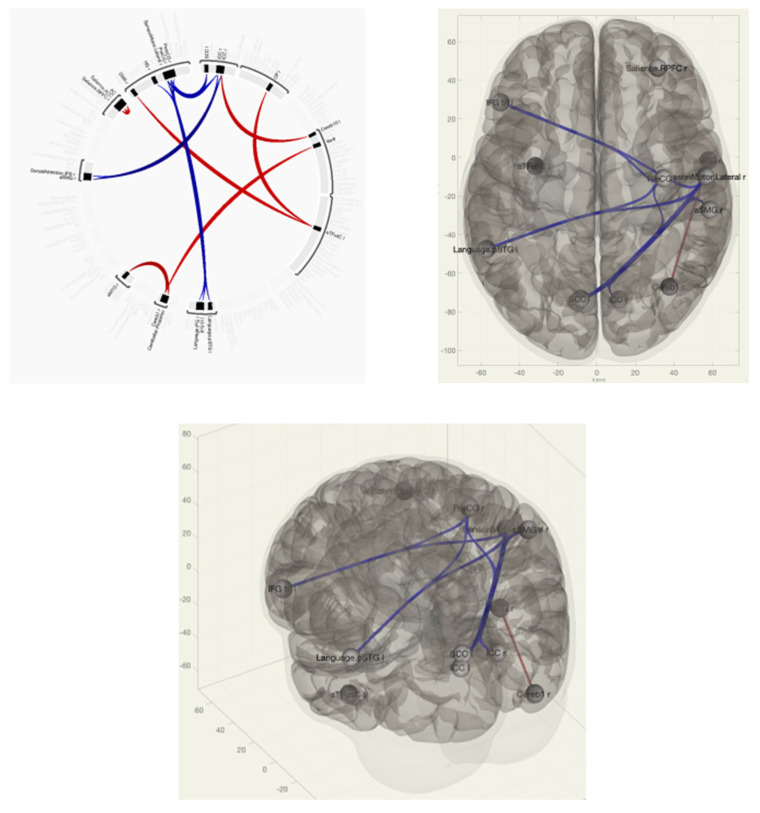
Functional connections of the brain of breast cancer survivors when comparing the group of VAS+/VAS− using the ROI-to-ROI method. The red color marks the positive contrast between groups, while the negative contrast is marked in blue.

**Figure 3:**
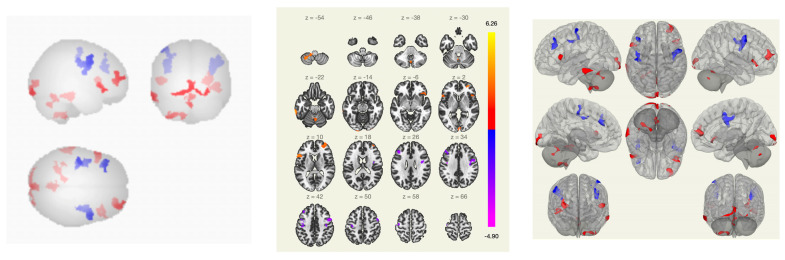
Functional connections of the brain of BCS when comparing the VAS–/VAS+ group using the seed-to-voxel method. The red color marks the positive contrast between groups, while the negative contrast is marked in blue.

In patients with VAS, when compared with the group without VAS, there was a predominance of functional connections between the pre- and postcentral gyri on the right and the Heschl gyrus on the right; between the inferior frontal gyrus on the left, the supra-spinal cortex, and the spur sulcus; between the spur sulcus and the dorsal network of attention; and between the anterior sections of the supramarginal gyrus ipsilateral right (Figure 2 and Figure 3).

Comparing the group of healthy volunteers and patients with vestibulo-atactic syndrome, stronger functional connections were revealed between the lateral visual network and the cerebellum, the right amygdala and the putamen, the thalamus and the temporal lobe, and between the inferior frontal gyrus and the cerebellum.

**Figure 4:**
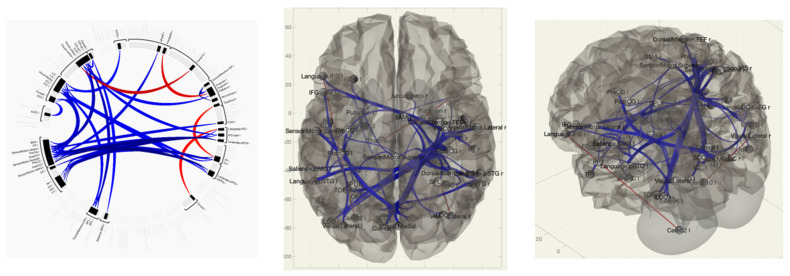
Functional connections of the brain of BCS when comparing the group of VAS + /healthy volunteers using the ROI-to-ROI method. By the red color is marked positive contrast between groups, negative contrast is marked in blue.

**Figure 5:**
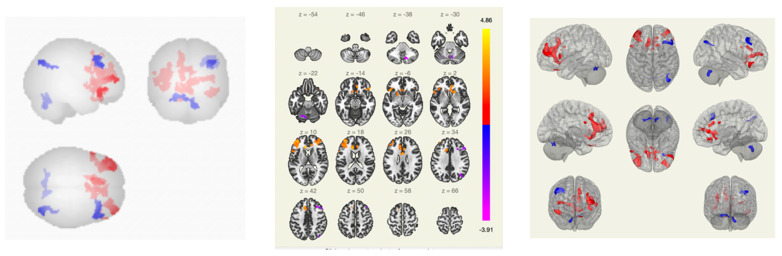
Functional connections of the brain of BCS when comparing the group of VAS+/healthy volunteers using the seed-to-voxel method. The red color marks the positive contrast between groups, while the negative contrast is marked in blue.

In patients with VAS, compared with the group of healthy volunteers, there was a weakening of functional connectivity between the lateral and superior somatosensory networks, the precentral gyri, the cortex of the calcarine fissure, and between the precentral gyrus and the Geschl gyrus; between the lingual gyrus, visual cortex and lateral occipital cortex, cunei, supra-spinal cortex, lateral and superior somatosensory networks, and precentral gyri; and between the middle temporal gyrus, inferior frontal gyrus, and lateral and medial visual networks (Figure 4 and Figure 5).

Along with this, in the group of patients with VAS, a greater strength of connections in the structures that provide visual control during movement, as well as connections from the centers of proprioception, which is necessary to correct the signs of postural and kinetic instability, was revealed. Thus, in patients with VAS, the processes of disorganization were noted, along with the formation and strengthening of inter- and intrahemispheric connections, which was expressed in the functional reorganization of neural networks.

## 4. Discussion

The study included BC patients with newly diagnosed manifestations of VAS in the long-term follow-up period after radical treatment of the tumor. Our study found statistically significantly higher serum levels of ICAM-1 molecules in the intergroup comparison, whereas PECAM-1, NSE, and lower levels of NR-2ab did not show significant differences in any of the studied subgroups. In addition, the results of fMRI revealed changes in the functional connectivity of the brain in the areas involved in the regulation of postural-tonic reflexes, the coordination of movements, and the regulation of balance, supporting the observations reported previously [25].

In our studies, we consider the systemic direct effect of chemotherapy on the CNS; however, studies have also shown damage to the peripheral nervous system, namely the development of polyneuropathy with a disorder of deep sensitivity associated with taxane therapy [51]. However, studies have described a representative cohort of cancer survivors, with imbalances in 50% of patients diagnosed with cancer. Notably, the vast majority (89.0%) of imbalances can be attributed to vestibular dysfunctions [52].

Evaluation of ICAM-1 and PECAM-1, potential biomarkers of neuronal and endothelial damage in BCS, demonstrated a significant increase in the levels of these molecules in patients with manifestations of vestibulo-atactic syndrome, which, in turn, suggests the leading role of endothelial dysfunction in the development of this pathology [25,53]. The obtained data are in line with a previously reported study by our group that showed a predictive role of ICAM-1 and PECAM-1 in an early onset of complications in BC survivors [54]. The increase in the NSE level in patients with VAS, compared with the other two groups, also confirms the data of our previous studies: due to the increase in NSE, it is possible to determine the damage to neurons and pathological activation of microglia, which are considered the leading mechanisms of cerebral disorders after breast cancer treatment [25]. As was shown by Schroeter et al., a specific association between serum NSE and cerebellar connectivity in the female human brain was revealed [55]. The cerebellum contains the majority (70–80%) of all neurons in the brain, and NSE expression is significantly higher in the cerebellar cortex [56]. NSE, as a marker of oxidative damage to the nervous system, may indicate a direct neurotoxic effect of chemotherapeutic agents. Moreover, the increase in the NSE levels may reflect the degree of pathological activation of neuroglia [25].

During acute (stroke, transient ischemic attack) or chronic cerebral ischemia, a cascade of pathological complexes is triggered, which causes excitatory molecules such as glutamate to enter the extracellular space, which, in turn, leads to the apoptosis of neurons due to their neurotoxic properties [57,58]. Therefore, detection of the NR-2 antibody could serve as biomarker to assess the neurotoxic effects of glutamate. In our study, we detected a nearly three-fold decrease in the serum levels of NR-2-ab (as compared to the group of healthy volunteers) (Table 3; Figure 1). These data are in line with previously reported data by Pospelova et al., which showed that the level of NR-2-ab among patients who underwent radical treatment for breast cancer and patients with chronic cerebral ischemia decreased, presumably due either to the immunosuppression caused by anticancer treatment or the depletion of glutamate-mediated mechanisms in the CNS [25].

Functional magnetic resonance imaging has been shown to be a useful tool for evaluating various neurological disorders (e.g., vestibular migraine and Alzheimer’s disease) and describing the changes in functional connectivity as a potential predictor of cognitive dysfunction [59,60]. Thus, Askren et al. demonstrated that the spatial variance in executive network activation, detected in women before chemotherapy for breast cancer, is able to predict the severity of cognitive impairment and fatigue following the treatment [61]. In our study, we observed the greater strength of connections in the structures responsible for visual control during movement, as well as connections from the centers of proprioception, which are necessary to correct the signs of postural and kinetic instability in patients with VAS. Changes in the sensorimotor network connectivity demonstrated the same tendency as was previously reported by Feng et al. for BC patients (*n =* 17) following chemotherapy [27]. Presumably, the increased functional connectivity may represent a compensation for chemotherapy to maintain normal neural activity. On the contrary, the decreased functional connectivity in other regions (i.e., the left and right frontoparietal network, anterior and posterior default mode network, self-referential network) may be associated with the reallocation of neural resources in information processing [27]. Another study by McDonald et al. showed regional reductions in the frontal, temporal, and parietal gray matter on VBM (voxel-based morphometry) [62]. Although there were no significant associations between brain imaging metrics and cognition, women with breast cancer showed increased activation during working memory processing that was not seen in control group. The reductions in gray matter volume in women with breast cancer and cortical thickness over time were revealed, which indicates the restructuring of the working networks of the brain [62]. Furthermore, de Ruiter et al. observed the reduction in gray matter volume (as measured by VBM) in the left lateral posterior parietal cortex, bilateral precuneus, left occipital cortex, and bilateral cerebellum overlapped with the hypoactive area in the left lateral posterior parietal cortex as measured with fMRI [12].

We were not able to find studies published to date investigating changes in functional connectivity in BCSs who had manifestations of vestibulo-atactic syndrome. At the same time, several studies have shown changes in the connectome in patients with cognitive disorders and depression, cerebellar ataxia, and multiple system atrophy [63,64,65]. These studies revealed changes in the connectogram in different areas of the brain, which indicates that there is no unambiguous opinion about the mechanisms of damage and the point of application of chemotherapy treatment.

In the present study, a correlation was shown between vestibulo-atactic syndrome and changes in functional connectivity in the areas responsible for coordination, balance, and the formation of postural-tonic reflexes in breast cancer survivors. Moreover, an increase in the serum levels of biomarkers (i.e., ICAM-1, PECAM-1, NSE) and changes in functional connectivity together might indicate a complex lesion of the central nervous system in VAS in patients in the long-term period following breast cancer treatment. Thus, an increase in ICAM-1 and PECAM-1 levels might indicate endothelial damage, while an increase in NSE levels may indicate neural damage (Figure 1). Endothelial dysfunction and chronic cerebral ischemia can lead to microstructural damage to the tracts of the white matter of the brain, as well as the violation of integrative functions, which, in turn, results in the clinical manifestation of VAS [54]. Furthermore, a reduced level of NR-2ab in patients may indicate immunosuppression as a result of radio- and chemotherapy, but further studies are of high importance to test this hypothesis. Presumably, the pathogenesis of VAS likely simultaneously involves the damage both to the neurons and vascular endothelium.

## 5. Conclusions

The development of new diagnostic algorithms based on clinical data, the identification of biomarkers, neuroimaging data for predicting CNS complications, and the detection of functional and organic disorders in women after breast cancer treatment are important aspects in rehabilitation and, accordingly, improving the quality of life, which is one of the directions of modern clinical oncology. In BCS patients with a clinical manifestation of VAS, changes in fMRI data that correspond to increases in the serum levels of ICAM-1, PECAM-1, and NSE may represent a pathogenetic pathway for CNS damage.

## Figures and Tables

**Table 1:** Data on the studied BCS patients and the control group, where VAS+ indicates patients with symptoms of vestibulo-atactic syndrome, and VAS− indicates patients without it.

	Totally	Age	Scope of Operation			Chemotherapy and/or Radiation Therapy		
			Madden (One or Bilateral)	Sectoral Resection	Subcutaneous Mastectomy with One-Stage Mammoplasty	Comprehensive Treatment	Combined Treatment	Surgery
VAS+	14	45.5 ± 5.19	12 (86%)	1 (7%)	1 (7%)	6 (43%)	7 (50%)	1 (7%)
VAS−	7	45.5 ± 4.1	7 (100%)	-	-	3 (43%)	4 (57%)	-
Control	17	44.25 ± 3.54	-	-	-	-	-	-

**Table 2:** Pulse sequence parameters of MRI.

	T2 BLADE	EP2D-DIFF MDDW	GRE Field Mapping	EP2D 120 REST	MPRAGE	T2 tse COR	TIRM
Repetition time/TR	4000 ms	4000 ms	400 ms	3000 ms	2300 ms	6100 ms	7000 ms
Echo time/TE	113 ms	92 ms	4.92 ms	30 ms	2.98 ms	93 ms	93 ms
FoV	220 mm	230 mm	192 mm	192 mm	256 mm	220 mm	220 mm
Slice thickness	5.0 mm	4.5 mm	3.0 mm	4.5 mm	1.2 mm	3.5 mm	4.0 mm
Voxel size x (mm), y (mm)	0.7 × 0.7	1.8 × 1.8	3.0 × 3.0	3.0 × 3.0	1.0 × 1.0	0.7 × 0.7	0.9 × 0.9
Study time	2:02	3:42	0:54	6:08	5:03	1:27	3:04

**Table 3:** The level of biomarkers of breast cancer survivors and the control group. Variables are represented by medians and ranges of values (Me [25 Percentile; 75 Percentile]).

Biomarker	Breast Cancer Survivors (*n* = 21)		Control (*n* = 17)	*p*-Value
ICAM-1 pg/mL	VAS+	654.7 [429; 1000]	230.2 [176; 311]	0.00001 *
	VAS−	551.9 [438; 706]		
PECAM-1 pg/mL	VAS+	115.3 [62; 191.8]	62.8 [30; 91]	0.920216
	VAS−	106 [61.4; 171.5]		
NSE pg/mL	VAS+	49.9 [8.82; 375]	15.5 [7.64; 36.04]	0.150443
	VAS−	20.3 [12.64; 70.87]		
NR-2-ab pg/mL	VAS+	0.5 [0.16; 1.06]	1.4 [0.57; 3.66]	0.646335
	VAS−	0.58 [0.23; 1.18]		

*—differences between the groups were significant at *p* < 0.05.

**Table 4:** Change in the activation level of functional connections, * T-indicator of functional connection in comparison of groups VAS+/VAS−.

Region of Study	Statistics, T *
occipital lobe, left	4.11
additional motor area, right	4.11
anterior fusiform gyrus, left	4.11
calcarine fissure, left	4.11
superior cerebellar peduncle, right	4.06
posterior lobe of the cerebellum, right	4.25
cerebellar vermis	4.06
Flocculus, left	4.18
precentral gyrus, right	−4.39
postcentral gyrus, right	−3.95
Geschl gyrus on the right	−3.90
inferior frontal gyrus, left	−4.39
supraspinal cortex, left	−3.89
calcarine fissure, left	−3.95
Dorsal Attention Network	−3.95

## Data Availability

Not applicable.

## Abbreviations

ADMN	anterior default mode network
BCS	breast cancer survivors
BOLD	blood oxygenation level dependent
CN	central network
CNS	central nervous system
DMN	Default Mode Network
fMRI	functional magnetic resonance imaging
GLOBOCAN	Global Cancer Observatory
ICAM-1	intercellular adhesion molecule 1
MDDW	multidirectional diffusion weighting
MPRAGE	Magnetization Prepared-Rapid Gradient Echo
NMDA	N-methyl-D-aspartate receptor
NR-2-ab	antibodies recognizing NR-2 subunit of NMDA receptor
NSE	neuron-specific enolase
PDMN	posterior default mode network
PECAM-1	platelet/endothelial cell adhesion molecule 1
SMN	sensorimotor network
TIRM	inversion recovery MRI pulse sequence
VAS	vestibulo-atactic syndrome
VBM	voxel-based morphometry
VN	visual network
WHO	World Health Organization
